# CD4^+^T cells do not mediate within-host competition between genetically diverse malaria parasites

**DOI:** 10.1098/rspb.2007.1713

**Published:** 2008-02-20

**Authors:** Victoria C Barclay, Lars Råberg, Brian H.K Chan, Sheila Brown, David Gray, Andrew F Read

**Affiliations:** 1 School of Biological Sciences, University of EdinburghEdinburgh EH9 3JT, UK; 2 Department of Animal Ecology, Lund University223 62 Lund, Sweden

**Keywords:** malaria, CD4^+^T cells, competition

## Abstract

Ecological interactions between microparasite populations in the same host are an important source of selection on pathogen traits such as virulence and drug resistance. In the rodent malaria model *Plasmodium chabaudi* in laboratory mice, parasites that are more virulent can competitively suppress less virulent parasites in mixed infections. There is evidence that some of this suppression is due to immune-mediated apparent competition, where an immune response elicited by one parasite population suppress the population density of another. This raises the question whether enhanced immunity following vaccination would intensify competitive interactions, thus strengthening selection for virulence in *Plasmodium* populations. Using the *P. chabaudi* model, we studied mixed infections of virulent and avirulent genotypes in CD4^+^T cell-depleted mice. Enhanced efficacy of CD4^+^T cell-dependent responses is the aim of several candidate malaria vaccines. We hypothesized that if immune-mediated interactions were involved in competition, removal of the CD4^+^T cells would alleviate competitive suppression of the avirulent parasite. Instead, we found no alleviation of competition in the acute phase, and significant enhancement of competitive suppression after parasite densities had peaked. Thus, the host immune response may actually be alleviating other forms of competition, such as that over red blood cells. Our results suggest that the CD4^+^-dependent immune response, and mechanisms that act to enhance it such as vaccination, may not have the undesirable affect of exacerbating within-host competition and hence the strength of this source of selection for virulence.

## 1. Introduction

Parasitic infections are often genetically diverse, with hosts concurrently infected by more than one genotype. Crowding, where pathogen populations within a host are suppressed by the presence of competitor strains, could affect the health and infectiousness of individual hosts as well as the evolution of medically relevant traits such as virulence and drug resistance (Read & Taylor 2001). For example, selection for increased virulence is expected when a slower growing parasite is outcompeted by a faster growing, more virulent parasite (Bremermann & Pickering 1983; van Baalan & Sabelis 1995; Frank 1996; Gandon *et al*. 2001; Alder & Losada 2002). Similarly, the relative fitness of drug-resistant strains, and hence their rate of spread in a population, could be substantially enhanced when co-infecting drug-sensitive competitors are removed by chemotherapy (e.g. Hastings 1997, 2003, 2006; Mackinnon & Hastings 1998; Hastings & D'Alessandro 2000; Mackinnon 2005). Analogous evolutionary processes could affect the rate of evolution of epitope variants in response to strain-specific vaccination (Lipsitch & Samore 2002; Read & Mackinnon 2008).

Infections with the human malaria parasite *Plasmodium falciparum* frequently consist of more than one genotype (Anderson *et al*. 2000; Awadalla *et al*. 2001; Jafari *et al*. 2004; Walliker *et al*. 2005), and a variety of epidemiological evidence is consistent with crowding (Daubersies *et al*. 1996; Mercereau-Puijalon 1996; Arnot 1998; Smith *et al*. 1999; Bruce *et al*. 2000; Hastings 2003; Talisuna *et al*. 2006). In the rodent malaria model *Plasmodium chabaudi* in laboratory mice, there is a strong relationship between parasite virulence and crowding such that more virulent strains have a competitive advantage (de Roode *et al*. 2003, 2005*a*,*b*; Bell *et al*. 2006).

A number of biological mechanisms may underlie competition between strains within hosts (Read & Taylor 2001). One of these is immune-mediated apparent competition (Holt 1977), where increasing densities of one pathogen population elicits a host response that suppresses the population of another. T cell-dependent immune-mediated competition has been demonstrated in *P. chabaudi* (Råberg *et al*. 2006): in nude mice, which cannot produce mature T cells, competition was less severe than in nude mice reconstituted with T cells. Because many malaria vaccines currently under trial are aimed at inducing T cell-dependent responses, that experiment raised the question of whether vaccination might exacerbate in-host competition and thus affect pathogen evolution, for instance by strengthening selection for competitive ability and hence virulence.

The effects of immunity on in-host competition are unlikely to be simple. The immune response to *Plasmodium* infection has both pathogen genotype-transcending (non-specific) and genotype-specific components. Protection is generally thought to become more specific during later stages of infection (Jarra & Brown 1989; Buckling & Read 2001; Mackinnon & Read 2003; Stevenson & Riley 2004; Martinelli *et al*. 2005; Cheesman *et al*. 2006). Thus, in contrast to non-specific immunity that could generate immune-mediated apparent competition, specific immunity could, in principle, act to alleviate competition (Råberg *et al*. 2006). Here, we extend the study by Råberg *et al*. (2006) by focusing on a specific subset of T cells, in order to further investigate the importance of immunity in determining competitive outcomes within hosts.

T cells can be divided into two major categories, CD4^+^ and CD8^+^ cells. It is well established from both experimental animal models and field studies in humans that the CD4^+^T cells play a pivotal role in the development of blood stage immunity to *Plasmodium* infection (Good & Doolan 1999; Pombo *et al*. 2002). They are initially required to produce cytokines that amplify the phagocytic and parasitocidal response of the innate immune response and later on to dampen this response to limit immunopathology. As the response becomes more adaptive they are required to help B cells produce antibodies that are essential for parasite clearance (Urban *et al*. 2005; Stephens & Langhorne 2006).

Since the CD4^+^T cells have been described as having such a crucial role in natural immunity to the blood stage of infection, and vaccine programmes strive to mimic and enhance this response (e.g. Stephens & Langhorne 2006), we have begun to investigate the specific role of these cells during competition in mixed infections of *P. chabaudi*. Specifically we looked at the acute phase of infection, where any interaction between the parasite and the host immune response could strongly influence host health (Urban *et al*. 2005). We chose two parasite genotypes that had been shown previously to differ in competitive ability and compared the extent of competition in immuno-competent and CD4^+^T cell-depleted mice. We hypothesized two possible scenarios: (i) if T cell-dependent immunity induces a non-specific response, then a numerically subdominant clone would experience a stronger immune response in a mixed infection than when on its own. Thus, competition should be eased in CD4^+^T cell-depleted mice. (ii) If the immune response is largely clone-specific and primarily elicited against the numerically dominant clone, then CD4^+^T cell depletion may exacerbate other forms of competition, such as competition for limited resources such as red blood cells.

## 2. Material and methods

### (a) Parasites and hosts

Isolates of *P. chabaudi* were originally collected from *Thamnomys rutilans* in the Central African Republic (Beale *et al*. 1978). These isolates have been genotyped and are stored as frozen stabilates in liquid nitrogen with subscript codes used to identify their position in the clonal history (Mackinnon & Read 1999). Two genotypes, AS_12062_ and DK_108_, were chosen based on their relative virulence and non-lethality. Pilot studies showed that clone DK achieved higher parasite densities when clone AS was absent than when AS was present. In contrast, clone AS was not competitively suppressed by DK. Hosts were inbred female C57BL/6JolaHsd mice aged six to eight weeks (Harlan England) maintained as described previously (de Roode *et al*. 2004).

### (b) Depletion of CD4^+^T lymphocytes *in vivo*

A rat monoclonal antibody, GK1.5, was used to deplete the CD4^+^T cells. A non-depleting rat monoclonal antibody of the same isotype (IgG 14131, Sigma) was used as a control. Experimental mice were injected intraperitoneally with 500 μg of the appropriate purified antibody in phosphate-buffered saline (PBS) 5 days before parasite challenge, and then with 250 μg antibody 4 days and 1 day before parasite challenge and weekly after challenge.

A fluorescence-activated cell sorter (FACS) was used to confirm CD4^+^T cell depletion. From the tail snip, 20 μl of blood was taken 1 day prior to injection with the appropriate antibodies. Single cell suspensions were made by removing red blood cells using Lympholyte according to the manufacturer's instructions (Cedarlane, Canada). Approximately 1×10^6^ cells were then transferred to a round-bottomed plate and resuspended in FACS buffer (PBS with 2% FCS with 0.05% sodium azide) before incubation for 20 min at 4°C with Allophycocyanin (APC)-labelled anti-CD4^+^ antibody (Pharmingen). The cells were washed three times in FACS buffer. Samples were collected on a FACS Calibre and 10 000 live events were collected for the majority of samples. FlowJo (TreeStar, CA) was used to analyse the data.

### (c) Experimental setup and sampling

Groups of five mice were treated with: (i) control antibodies and challenged with 10^6^ AS parasites, (ii) control antibodies and challenged with 10^6^ DK parasites, (iii) control antibodies and challenged with 10^6^ AS and 10^6^ DK, (iv) anti-CD4 antibodies and challenged with 10^6^ AS, (v) anti-CD4 antibodies and challenged with 10^6^ DK, and (vi) anti-CD4 antibodies and challenged with 10^6^ AS and 10^6^ DK.

Parasites were delivered by intraperitoneal injection. We used the same dose of each genotype in single and mixed infections (rather than the same total dose in single and mixed infections) because the aim of the study was to compare the performance of a genotype when it is on its own, with its performance when it is in a mixed infection. A twofold difference in infective dose has negligible effects on the population dynamics of the parasite (Timms *et al*. 2001). In addition, we included two extra control groups, each of two mice that were not challenged with malaria, one group treated with anti-CD4 antibodies and another group with control antibodies. These mice were used to check whether CD4^+^T cell depletion was continuous throughout the experiment, as the number of peripheral T cells was lower than normal during the acute stage of disease (Hviid *et al*. 1997).

During the course of infection, we measured body weights and took blood samples from the tail to make Giemsa-stained blood smears and to estimate RBC density (by flow cytometry; Beckman Coulter) and for genotype-specific real-time quantitative PCR (qPCR) assays.

One mouse died during the experiment (CD4^+^T cell-depleted, mixed infection) and was included in the analyses only where possible. For unknown reasons, two infections (both non-depleted, a DK-only and an AS-only) achieved a peak parasite density two orders of magnitude lower than all others, and these were excluded from all the analyses.

### (d) Quantitative PCR

Samples were taken in the morning as this is the stage when most parasites are in the ring or early trophozoite stage in the peripheral blood, when parasite ploidy is stable (de Roode *et al*. 2004). From each mouse, 5 μl of tail blood was taken and added to 100 μl of citrate saline on ice. Samples were subsequently pelleted by centrifugation and the citrate saline was removed. Blood was stored at −80°C until required. DNA extraction was performed using the BloodPrep kit (Applied Biosystems) on the ABI PRISM 6100 Nucleic Acid prep-station according to the manufacturer's instructions. DNA was eluted in a total volume of 200 μl and stored at −80°C until quantification. Genotype-specific qPCR was performed as described previously (Bell *et al*. 2006) with the addition of the DK-specific reverse primer: 5*′*-AGG CAT GTT TTG CAC ACA ATG A-3*′*.

### (e) Trait definition and statistical analyses

We define competitive suppression to be a reduction of parasite numbers when another clone is present, which we tested for by comparing the performance of a clone in single and mixed infections. Performance was measured as the clonal density summed over a defined time period. *Plasmodium chabaudi* has a 24 hour replication cycle, so the total number of parasites present in any period can be estimated by summing the daily parasite counts. Thus, to test whether competitive suppression was CD4^+^T cell mediated, we asked, for each clone, whether the magnitude of any competitive suppression differed between intact control and CD4^+^T cell-depleted hosts; that is, whether there was a statistical interaction between immune treatment (intact control versus CD4^+^T cell-depleted hosts) and infection type (single versus mixed).

The effects of competition and CD4^+^ depletion on the performance of individual clone and red blood cell density were first examined by using general linear models (GLM) in the statistical package Minitab (release 14, Minitab, Inc.,). For GLM analysis, response variables included mean total parasite density and mean RBC density, with initial RBC as a covariate. Explanatory variables for GLM included CD4^+^ depletion (depleted or intact control) and competition (clone alone or in mixed infection). Maximal models (response variable = CD4^+^ depletion + competition + all higher order interactions) were tested in the first instance, and minimal models were obtained by dropping non-significant terms successively, beginning with highest order interactions, to obtain the significant minimal model. Second, we used repeated-measures analyses that take into account the importance of day post-infection. These analyses were performed as described by Råberg *et al*. (2006) using the statistical package SAS v. 9.1 (SAS Institute 1999, SAS OnlineDoc. v. 8. SAS Institute, Cary, NC). Briefly, the analyses were performed with PROC MIXED, using the REPEATED statement (subject=mouse), the Satterthwaite approximation of the denominator degrees of freedom, and the autoregressive covariance structure AR(1). Within each treatment group, the peak day varied ±2 days, presumably as a result of slight differences in inoculation dose. To control for this variation, we centred the peak day at the median peak day within each treatment group. All density data were transformed using [log(density +10)].

## 3. Results

**Figure 1:**
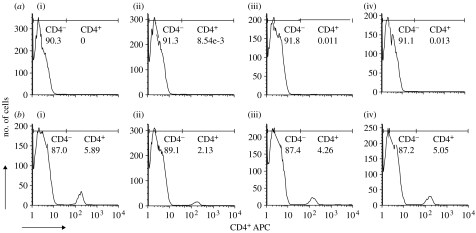
FACS plots of number of cells analysed and percentage of CD4^+^T cells in (*a*) CD4^+^T cell-depleted and (*b*) intact control mice. Percentage of the CD4^+^T cells was analysed: (i) 1 day before parasite challenge and (ii–iv) once a week throughout the experiment. Each graph is a representative of one mouse from either the CD4^+^T cell-depleted or immunocompetent control group.

**Figure 2:**
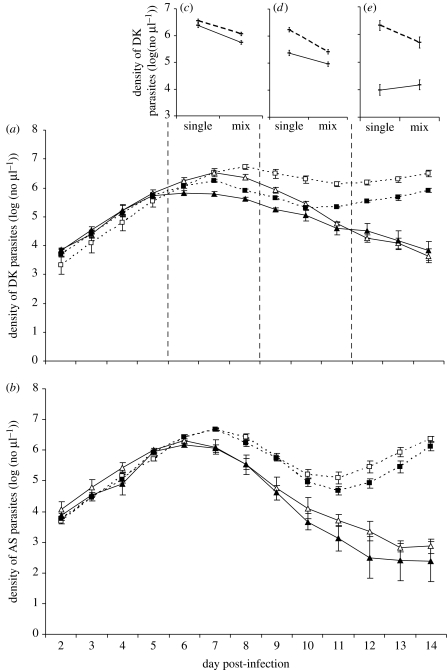
Parasite densities through time of (*a*) clone DK and (*b*) clone AS, and (*c*–*e*) average densities of DK during the periods denoted by the vertical dashed lines. Asexual density from qPCR of CD4^+^T cell-depleted mice and intact control (normal) mice with single infections in CD4^+^T cell-depleted mice and mixed infections in intact control (normal) mice. Mean densities (±1 s.e.m.) were calculated from all mice that were alive on the respective day of sampling (up to five). The interaction plots (*c*–*e*) show the total numbers of DK parasites when the competitor clone AS is absent (single infection) or present (mixed infection) in CD4^+^-depleted mice (dashed lines) and normal animals (solid lines). Competition×depletion interactions: (*c*) *F*_1,16_=1, *p*=0.33; (*d*) *F*_1,17_=5.11, *p*=0.037; and (*e*) *F*_1,14_=4.57, *p*=0.0499 (open squares, CD4^+^-depleted single; open triangles, intact single; filled squares, CD4^+^-depleted mixed; filled triangles, intact mixed).

Mice treated with the anti-CD4^+^T cell antibody were successfully depleted of CD4^+^T cells, both prior to parasite challenge and during the whole course of the experiment (figure 1). The CD4^+^T cell depletion resulted in more parasites of both clones (figure 2*a*,*b*; table 1).

### (a) Clone DK

As found with other pairs of clones (de Roode *et al*. 2005*a*,*b*; Bell *et al*. 2006), here we found that the relatively avirulent clone was competitively suppressed by the more virulent clone, with DK achieving lower parasite densities when AS was present than when it was absent (figure 2*a*; table 1). However, the extent of competitive suppression of clone DK was similar regardless of CD4^+^T cell depletion (table 1; depletion×competition interaction, n.s.). Thus, there was no evidence that the competitive suppression of the total number of DK parasites present in an infection was mediated by CD4^+^T cell-dependent immunity.

However, repeated-measures analysis of the period where CD4^+^T cell depletion affected parasite densities (day 6 onwards) showed a weak but significant three-way depletion×competition×day interaction (table 2). To investigate this further, and following Råberg *et al*. (2006), we divided the data into three parts, days 6–8, 9–11 and 12–14, and repeated the analyses with each of these (figure 2*a*,*c*–*e*; table 3). During each of these time periods, there were significant depletion×competition or depletion×competition×day interactions. Inspection of figure 2*a*,*c* shows that the three-way interaction in the first period is a very weak effect from which it is difficult to conclude much, given the rapid alterations in infection kinetics during that period caused by depletion. In the other two periods, there are significant competition×depletion interactions (figures 2*d*,*e*; table 3), with more severe competitive suppression in CD4^+^T cell-depleted mice than in control mice. Thus, there was no evidence that competitive suppression is CD4^+^T cell mediated: once the initial wave of parasitaemia began to subside, competitive suppression was exacerbated rather than alleviated in CD4^+^-depleted mice.

### (b) Clone AS

There was no evidence of competitive suppression of AS by DK, irrespective of the immune treatment (figure 2*b*; table 1). Repeated-measures analysis from day 6 onwards, when CD4^+^ depletion had an effect, revealed no evidence of interactions between depletion and competition (table 2). However, for comparison with the analysis of clone DK, we repeated the same analyses for AS on days 6–8, 9–11 and 12–14 (table 4). In none of these time periods was there any evidence of competitive suppression (in all cases, competition main effect and depletion×competition, *p*>0.15).

### (c) Red blood cells

**Figure 3:**
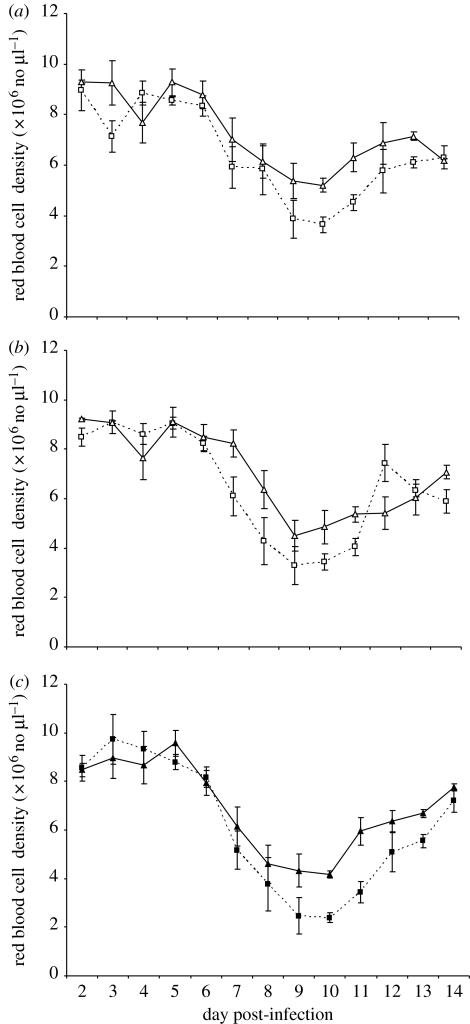
Mean red blood cell densities (±1 s.e.m) over time. (*a*) CD4^+^T cell-depleted and intact mice infected with DK (squares, depleted single; triangles, intact single), (*b*) depleted and intact mice infected with AS (squares, depleted single; triangles, intact single), and (*c*) depleted and intact mice infected with DK and AS (squares, depleted mixed; triangles, intact mixed).

Red blood cell density over time for the different treatment groups are shown in figure 3. Uninfected red blood cells form an important resource for malaria parasites. To assess whether the potential for competition over this resource differed between CD4^+^-depleted and intact control mice, we compared the red blood cell densities in mice with mixed infections. Repeated-measures analysis of days 6–14 revealed that CD4^+^-depleted mice had significantly lower red blood cell densities during this time period (*F*_1,34.7_=4.35, *p*=0.045; figure 3). There was also a significant depletion×day interaction (*F*_8,94.1_=2.58, *p*=0.014). Separate analyses of days 6–8, 9–11 and 12–14 showed that the difference in RBC density was most pronounced during days 9–14 (table 5).

## 4. Discussion

We found no evidence that the CD4^+^T cells enhanced competition during mixed genotype infections with *P. chabaudi* (figure 2*a*; table 1). Specifically, during the peak stages of acute infection (days 6–8) suppression was independent of the CD4^+^T cells (figure 2*c*; table 3). After the peak of infection (day 9+), the CD4^+^T cells acted to alleviate competition such that upon their removal competitive suppression was enhanced (figure 2*d*,*e*; table 3). In addition, the presence of CD4^+^T cells did not cause suppression of the dominant genotype (figure 2*b*; table 4).

The immune response to *Plasmodium* infection has both pathogen genotype-transcending (non-specific) and genotype-specific components, with protection becoming more specific during later stages of infection (see §1). Here, we found that after the peak of acute infection (day 9 onwards), there was no competitive suppression of DK parasites in intact control mice; whereas in CD4^+^T cell-depleted mice, there was still evidence of competition (figure 2*d*,*e*; table 3). Both clones did better in depleted mice, probably owing to an impaired early antibody production through lack of T cell help and possibly the reduced recruitment and activation of macrophages for the uptake of infected cells. Thus, in normal hosts, a largely clone-specific adaptive immune response towards a numerically dominant genotype may act to alleviate competition by regulating clonal populations and limiting other forms of competition, e.g. competition for red blood cells.

During the peak of infection (days 6–8), competition was CD4^+^T cell independent so that the extent of competitive suppression of clone DK was similar in intact control and CD4^+^T cell-depleted mice (figure 2*a*,*c*; table 3). A number of biological mechanisms could be the proximate cause of competitive suppression during the peak of infection. First, there may be direct interference between two infecting strains. This has not yet been demonstrated in any parasites, but pathogenic bacteria can produce allelopathic substances that actively suppress competitors (Riley & Gordon 1999), and competing viruses can produce interference molecules (Hart & Cloyd 1990). Second, the competition may be influenced by non-specific components of the innate immune response (CD4^+^T cell independent). Third, there may be competition for resources as genotypes infecting mice simultaneously must divide the available red blood cells and other resources such as blood glucose between them (Hellriegel 1992; Hetzel & Anderson 1996; de Roode *et al*. 2005*a*; Gurarie *et al*. 2006). The CD4^+^T cell-depleted mice were more anaemic than control mice (figure 3; table 5), so that if red cells are limiting, there is more potential for competition for that resource in depleted mice. Mathematical models have suggested that during mixed infection, the proximate cause of competitive advantage may be attributable to an earlier and wider red blood cell preference of dominant genotypes (Hellriegel 1992; Gravenor *et al*. 1995; McKenzie & Bossert 1997; Jakeman *et al*. 1999; Mason & McKenzie 1999; McQueen *et al*. 2004; Antia *et al*. submitted). Because these predictions are based on the data from the rodent malaria model, they could be tested directly by transferring red blood cells of different ages into a single mouse and determine their loss following infection, or indirectly by measuring competition in untreated mice and mice treated with erythropoietin (Suzuki *et al*. 2006).

Our conclusion that competition is not CD4^+^T cell mediated apparently contradicts the recent finding of T cell-mediated apparent competition (Råberg *et al*. 2006). In that study, the authors looked at mixed infections with *P. chabaudi* in nude mice (which lack the ability to produce mature T cells) and compared the extent of competition with that in nude mice reconstituted with T cells. There was still pronounced competition in all animals, but there was some alleviation of competitive suppression in nude mice towards the end of the acute phase of infection, when the initial wave of parasitaemia was waning. This period corresponds roughly to days 9–14 in figure 2. A number of experimental differences could explain the contrasting results of Råberg *et al*. (2006) and the present study. First, different mouse strains were used in the two studies and host genotype has previously been shown to quantitatively affect the outcome of competition (de Roode *et al*. 2004). Second, different pairs of clones were used and *P. chabaudi* clone can induce different levels of strain-specific immunity (Cheesman *et al*. 2006). Third, there was a difference in the method used to modulate T cell-dependent immunity. Nude mice lack the ability to produce any mature T cells, including both CD4^+^ and CD8^+^T cells. The role of CD8^+^T cells during malaria infection in mice is still unclear (Lamb *et al*. 2006), but it could be that they are involved in the relatively small component of competition that was shown to be immune-mediated competition in reconstituted nude mice (Råberg *et al*. 2006). In addition, the repertoire of serum antibodies (including both natural antibodies and antigen elicited antibodies) in the CD4^+^T cell-depleted mice will be different from that in nude mice. Nude mice grow up producing only T cell-independent antibodies, while in the CD4^+^T cell-depleted mice there will be both T cell-independent and persisting T cell-dependent antibodies (produced by existing plasma cells in the bone marrow) and these may cross react with the parasite.

Taken together, the present study and that of Råberg *et al*. (2006) show that the effect of T cell-dependent immunity on competition is relatively weak, and may be either positive or negative depending on specific details of host and parasite. Rather than further dissection of any immune mechanism-mediating competition, one could use this malaria model system to look at the strength of competition in hosts immunized by a variety of different candidate vaccines towards the blood stage of infection. Meanwhile, the result we report here suggest that vaccines that enhance CD4^+^-dependent immunity will not increase the selection in favour of virulence arising from in-host competition.

## Figures and Tables

**Table 1:** GLM analyses of the effects of CD4^+^T cell depletion, competition (presence/absence of co-infecting clone) and their interaction on the total number of parasites during the first two weeks of infection for parasite clone DK and AS.

	DK days 2–14			AS days 2–14		
		
effect	d.f.	*F*	*p*	d.f.	*F*	*p*
CD4^+^ depletion	1,15	82.7	<0.001	1,15	93.36	<0.000
competition	1,15	84.2	<0.001	1,15	2.05	0.17
CD4^+^ depletion×competition	1,15	0.6	0.74	1,15	0.94	0.94

**Table 2:** Repeated-measures analyses of the effects of CD4^+^ depletion, competition (presence/absence of co-infecting clone) and day post-infection on the daily densities of the two parasite clones DK and AS for days 6–14 post-infection.

	DK days 6–14			AS days 6–14		
		
effect	d.f.	*F*	*p*	d.f.	*F*	*p*
CD4^+^ depletion	1,16	74.52	<0.0001	1,17	51.58	<0.0001
competition	1,16	17.92	0.0006	1,17	1.86	0.19
day	8,101	30.8	<0.0001	8,108	40.85	<0.0001
CD4^+^ depletion×competition	1,16	2.65	0.12	1,17	0.02	0.88
CD4^+^ depletion×day	8,101	22.35	<0.0001	8,108	15.67	<0.0001
competition×day	8,101	3.67	0.0009	8,108	0.91	0.51
CD4^+^ depletion× competition×day	8,101	2.10	0.042	8,108	0.47	0.87

**Table 3:** Repeated-measures analyses of the effects of CD4^+^ depletion, competition (presence/absence of co-infecting clone) and day post-infection on the daily parasite density of clone DK for days 6–8, 9–11 and 12–14 post-infection.

	days 6–8			days 9–11			days 12–14		
			
effect	d.f.	*F*	*p*	d.f.	*F*	*p*	d.f.	*F*	*p*
CD4^+^ depletion	1,16	12.61	0.0026	1,17	54.79	<0.0001	1,14	105.95	<0.0001
competition	1,16	62.31	<0.001	1,17	47.53	<0.0001	1,14	1.33	0.26
day	2,29	15.32	<0.001	2,30	39.12	<0.0001	2,28	2.22	0.12
CD4^+^ depletion× competition	1,16	1	0.33	1,17	5.11	0.037	1,14	4.57	0.049
CD4^+^ depletion× day	2,29	2.88	0.071	2,30	11.87	0.0002	2,28	23.78	<0.0001
competition×day	2,29	12.34	0.0001	2,30	2.32	0.11	2,28	0.64	0.53
CD4^+^ depletion× competition×day	2,29	3.78	0.034	2,30	2.3	0.11	2,28	0.17	0.84

**Table 4:** Repeated-measures analyses of the effects of CD4^+^ depletion, competition (presence/absence of co-infecting clone) and day post-infection on the daily parasite density of clone AS for days 6–8, 9–11 and 12–14 post-infection.

	days 6–8			days 9–11			days 12–14		
			
effect	d.f.	*F*	*p*	d.f.	*F*	*p*	d.f.	*F*	*p*
CD4^+^ depletion	1,14	17.58	0.0008	1,15	31.83	<0.001	1,14	58.71	<0.001
competition	1,14	0.3	0.58	1,15	2.08	0.16	1,14	1.6	0.22
day	2,28	39.82	<0.001	2,29	89.06	<0.001	2,27	6.52	0.0049
CD4^+^ depletion× competition	1,14	0.01	0.91	1,15	0.11	0.74	1,14	0.03	0.86
CD4^+^ depletion ×day	2,28	12.17	0.0001	2,29	3.03	0.06	2,27	15.34	<0.001
competition×day	2,28	0.22	0.8	2,29	3.13	0.058	2,27	0.76	0.47
CD4^+^ depletion×competition×day	2,28	1.20	0.31	2,29	0.08	0.92	2,27	0.70	0.50

**Table 5:** Repeated-measures analyses of the effects of CD4^+^ depletion and day post-infection on the mean red blood cell density in mixed infections during days 6–8, 9–11 and 12–14.

	days 6–8			days 9–11			days 12–14		
			
effect	d.f.	*F*	*p*	d.f.	*F*	*p*	d.f.	*F*	*p*
CD4^+^ depletion	1,21	1.77	0.14	1,23	10.75	0.0033	1,19	4.57	0.045
day	2,36	42.18	<0.001	2,37	11.98	<0.0001	2,38	0.33	0.72
CD4^+^ depletion× day	2,26	3.18	0.05	2,37	0.65	0.52	2,28	0.08	0.92

## References

[bib1] Alder F.R, Losada J.M, Dieckmann U, Metz J.A.J, Sabelis M.W, Sigmund K (2002). Super-and co-infection: filling the range. Adaptive dynamics of infectious diseases: in pursuit of virulence management.

[bib2] Anderson T.J.C (2000). Microsatellite markers reveal a spectrum of population structures in the malaria parasite *Plasmodium falciparum*. Mol. Biol. Evol.

[bib3] Antia, R., Yates, A. & De Roode, J. C. Submitted. Virulence and competition in malaria infections.

[bib4] Arnot D (1998). Clone multiplicity of *Plasmodium falciparum* infections in individuals exposed to variable levels of disease transmission. Trans. R. Soc. Trop. Med. Hyg.

[bib5] Awadalla P, Walliker D, Babiker H.A, Mackinnon M.J (2001). The question of *Plasmodium falciparum* population structure. Trends Parasitol.

[bib5a] Beale G.H, Walliker D, Carter R, Killick-Kendrick R, Peters W (1978). Rodent malaria.

[bib6] Bell A.S, de Roode J.C, Sim D, Read A.F (2006). Within-host competition in genetically diverse malaria infections: parasite virulence and competitive success. Evolution.

[bib7] Bremermann H.J, Pickering J (1983). A game-theoretical model of parasite virulence. J. Theor. Biol.

[bib8] Bruce M.C, Donnelly C.A, Alpers M.P, Galinski M.R, Barnwell J.W, Walliker D, Day K.P (2000). Cross-species interactions between malaria parasites in humans. Science.

[bib9] Buckling A, Read A.F (2001). The effect of partial host immunity on the transmission of malaria parasites. Proc. R. Soc. B.

[bib10] Cheesman S, Raza A, Carter R (2006). Mixed strain infections and strain-specific protective immunity in the rodent malaria parasite *Plasmodium chabaudi chabaudi* in mice. Infect. Immunol.

[bib11] Daubersies P, Sallenave-Sales S, Magne S, Trape J.-F, Contamin H, Fandeur T, Rogier C, Mercereau-Puijalon O, Druilhe P (1996). Rapid turnover of *Plasmodium falciparum* populations in asymptomatic individuals living in a high transmission area. Am. J. Trop. Med. Hyg.

[bib12] de Roode J.C, Read A.F, Chan H.K, Mackinnon M.J (2003). Rodent malaria parasites suffer from the presence of con-specific clones in three-clone *Plasmodium chabaudi* infections. Parasitology.

[bib13] de Roode J.C, Culleton R, Cheesman S.J, Carter R, Read A.F (2004). Host heterogeneity is a determinant of competitive exclusion or coexistence in genetically diverse malaria infections. Proc. R. Soc. B.

[bib14] de Roode J.C, Helinski M.E.H, Anwar M.A, Read A.F (2005a). Dynamics of multiple infection and within-host competition in genetically diverse malaria infections. Am. Nat.

[bib15] de Roode J.C (2005b). Virulence and competitive ability in genetically diverse malaria infections. Proc. Natl Acad. Sci. USA.

[bib16] Frank S.A (1996). Models of parasite virulence. Q. Rev. Biol.

[bib17] Gandon S, Mackinnon M.J, Nee S, Read A.F (2001). Imperfect vaccines and the evolution of pathogen virulence. Nature.

[bib18] Good M.F, Doolan D.L (1999). Immune effector mechanisms in malaria. Curr. Opin. Immunol.

[bib19] Gravenor M.B, McLean A.R, Kwiatkowski D (1995). The regulation of malaria parasitemia–parameter estimates for a population-model. Parasitology.

[bib20] Gurarie D, Zimmerman P.A, King C.H (2006). Dynamic regulation of single- and mixed-species malaria infection: insights to specific and non-specific mechanisms of control. J. Theor. Biol.

[bib20a] Hart A.R, Cloyd M.W (1990). Interference patterns of human immunodeficiency viruses HIV-1 and HIV-2. Virology.

[bib20b] Hastings I.M (1997). A model for the origins and spread of drug-resistant malaria. Parasitology.

[bib21] Hastings I.M (2003). Malaria control and the evolution of drug resistance: an intriguing link. Trends Parasitol.

[bib20c] Hastings I.M (2006). Complex dynamics and stability of resistance to antimalarial drugs. Parasitology.

[bib21a] Hastings I.M, D'Alessandro U (2000). Modelling a predictable disaster: the rise and spread of drug-resistant malaria. Parasitol. Today.

[bib22] Hellriegel B (1992). Modelling the immune response to malaria with ecological concepts: short-term behaviour against long-term equilibrium. Proc. R. Soc. B.

[bib23] Hetzel C, Anderson R (1996). The within-host cellular dynamics of blood-stage malaria: theoretical and experimental studies. Parasitology.

[bib24] Holt R.D (1977). Predation, apparent competition, and the structure of prey communities. Theor. Popul. Biol.

[bib25] Hviid L, Kurtzhals J.A, Goka B.Q, Oliver-Commey J.O, Nkrumah F.K, Theander T.G (1997). Rapid reemergence of T cells into peripheral circulation following treatment of severe and uncomplicated *Plasmodium falciparum* malaria. Infect. Immunol.

[bib26] Jafari S, Le Bras J, Bouchaud O, Durand R (2004). *Plasmodium falciparum* clonal population dynamics during malaria treatment. J. Infect. Dis.

[bib27] Jakeman G, Saul A, Hogarth W, Collins W (1999). Anaemia of acute malaria infections in non-immune patients primarily results from destruction of uninfected erythrocytes. Parasitology.

[bib28] Jarra W, Brown K.N (1989). Invasion of mature and immature erythrocytes of CBA/ca mice by a cloned line of *Plasmodium chabaudi chabaudi*. Parasitology.

[bib30] Lamb T.J, Brown D.E, Potocnik A.J, Langhorne J (2006). Insights into the immunopathogenesis of malaria using mouse models. Expert Rev. Mol. Med.

[bib32] Lipsitch M, Samore M.H (2002). Antimicrobial use and antimicrobial resistance: a population perspective. Emerg. Infect. Dis.

[bib33a] Mackinnon M.J (2005). Drug resistance models for malaria. Acta Tropica.

[bib33b] Mackinnon M.J, Hastings I.M (1998). The evolution of multiple drug resistance in malaria parasites. Trans. R. Soc. Trop. Med. Hyg.

[bib34] Mackinnon M.J, Read A.F (1999). Genetic relationships between parasite virulence and transmission in the rodent malaria *Plasmodium chabaudi*. Evolution.

[bib33] Mackinnon M.J, Read A.F (2003). The effects of host immunity on virulence transmission relationships in the rodent malaria parasite *Plasmodium chabaudi*. Parasitology.

[bib35] Martinelli A, Cheesman S, Hunt P, Culleton R, Raza A, Mackinnon M, Carter R (2005). A genetic approach to the de novo identification of targets of strain specific immunity in malaria parasites. Proc. Natl Acad. Sci. USA.

[bib36] Mason D.P, McKenzie F.E (1999). Blood-stage dynamics and clinical implications of mixed *Plasmodium vivax*–*Plasmodium falciparum* infections. Am. J. Trop. Med. Hyg.

[bib37] McKenzie F.E, Bossert W.H (1997). The dynamics of *Plasmodium falciparum* blood-stage infection. J. Theor. Biol.

[bib38] McQueen P.G, McKenzie F.E, Singer B.H (2004). Age-structured red blood cell susceptibility and the dynamics of malaria infections. Proc. Natl Acad. Sci. USA.

[bib39] Mercereau-Puijalon O (1996). Revisiting host/parasite interactions: molecular analysis of parasites collected during longitudinal and cross-sectional surveys in humans. Parasite Immunol.

[bib40] Pombo D.J (2002). Immunity to malaria after administration of ultra-low doses of red cells infected with *Plasmodium falciparum*. Lancet.

[bib41] Råberg L, de Roode J.C, Bell A.S, Stamou P, Gray D, Read A.F (2006). The role of immune-mediated apparent competition in genetically diverse malaria infections. Am. Nat.

[bib42] Read A.F, Mackinnon M.J, Stearns S.C, Koella J (2008). Pathogen evolution in a vaccinated world. Evolution in health and disease.

[bib42a] Read A.F, Taylor L.H (2001). The ecology of genetically diverse infections. Science.

[bib42b] Riley M.A, Gordon D.M (1999). The ecological role of bacteriocins in bacterial competition. Trends Microbiol.

[bib43] Smith T, Felger I, Kitua A, Tanner M, Beck H.P (1999). Dynamics of multiple *Plasmodium falciparum* infections in infants in a highly endemic area of Tanzania. Trans. R. Soc. Trop. Med. Hyg.

[bib44] Stephens R, Langhorne J (2006). Priming CD4^+^T cells and development of CD4^+^T cell memory; lessons for malaria. Parasite Immunol.

[bib45] Stevenson M.M, Riley E.M (2004). Innate immunity to malaria. Nat. Rev. Immunol.

[bib46] Suzuki M, Ohneda K, Hosoya-Ohmura S, Tsukamoto S, Ohneda O, Philipsen S, Yamamoto M (2006). Real-time monitoring of stress erythropoiesis *in vivo* using Gata1 and beta-globin LCR luciferase transgenic mice. Blood.

[bib47] Talisuna A.O, Erhart A, Samarasinghe S, Van Overmeir C, Speybroeck N, D'Alessandro U (2006). Malaria transmission intensity and the rate of spread of chloroquine resistant *Plasmodium falciparum*: why have theoretical models generated conflicting results?. Infect. Gen. Evol.

[bib48] Timms R, Colegrave N, Chan B.H.K, Read A.F (2001). The effect of parasite dose on disease severity in the rodent malaria *Plasmodium chabaudi*. Parasitology.

[bib49] Urban B.C, Ing R, Stevenson M.M (2005). Early interactions between blood-stage plasmodium parasites and the immune system. Curr. Top. Microbiol. Immunol.

[bib50] van Baalan M, Sabelis M.W (1995). The dynamics of multiple infection and the evolution of virulece. Am. Nat.

[bib51] Walliker D, Hunt P, Babiker H (2005). Fitness of drug-resistant malaria parasites. Acta Tropica.

